# From ‘quarterback’ to ‘coach’: the policy implications of family physicians’ evolving role in team-based care

**DOI:** 10.1186/s12875-026-03202-y

**Published:** 2026-01-31

**Authors:** Myles Leslie, Anita McDonald

**Affiliations:** School of Public Policy and Cumming School of Medicine, University of Calgary, Calgary, AB Canada

**Keywords:** Primary health care, Access, Team-Based care, Nurse

## Abstract

**Background:**

Project Colourful, an innovative model for delivering team-based care and improving access to family physicians at a specific Canadian clinic, provided a window on how physician-led primary care teams are evolving. Colourful allowed an action research team to draw out broadly applicable policy lessons as family physicians moved from exclusively ‘quarterbacking’ direct patient care to also taking on a more off-field role as ‘coaches’ of registered nurses.

**Methods:**

Participant observations (*n* = 12) of Colourful’s design and implementation were supplemented with semi-structed interviews (*n* = 11) that focused on the project’s origins; plans for scaling; and interprofessional teamwork issues. Transcribed interviews were analysed using an interpretive descriptive approach.

**Results:**

The shift from quarterback to coach requires not only payment reform, but cultural change and training for physicians and other team members. Specific training here focuses on developing human resource skills and capacities that span: hiring, scope-of practice education, team dynamics, team member retention, and change management.

**Conclusions:**

Key support, resource, and governance considerations for policy makers looking to scale care models like Colourful include education to: flatten traditional interprofessional hierarchies; enable the communication required for successful teamwork; as well as, bolster the HR skills of physician coaches; ensure an optimized interprofessional mix; and empower team members to renegotiate scopes of practice at clinical and provincial levels.

## Background

 Canadians’ access to family physicians (FPs) has become an acute policy challenge [1–3]. This long simmering crisis in access [4–7] has evolved alongside decades of policy innovation focused on creating and supporting FP-led teams of primary health care (PHC) providers [5, 8–10]. With concerns expressed about the health system’s capacity to recruit, educate, and retain FPs [11–13], questions about the viability of FP-led teams and the role physicians play on them come to the fore. In a funding, clinical, and North American political/cultural environment where FPs have been ‘quarterbacking’ [14–17] what does a shift towards ‘coaching’ involve? If there is indeed a move from on-the-field leadership towards from-the-sidelines oversight happening, what does it look like practically and are present policy supports well adapted to ensuring the success of these new teams?

To answer these research questions, this paper draws on qualitative observation and interview data collected as a team of PHC providers in Alberta, Canada embraced a new ‘coaching’ role for FPs. On the one hand it is an account of the specifics of the Alberta providers’ experiences as they designed and executed “Project Colourful” (Colourful)^1^ to respond to their local iteration of Canada’s access-to-PHC crisis. On the other, the paper highlights broadly applicable policy considerations as the nature of team-based care and the activities and competencies of FPs are changing. In this sense we have sought answers to the broad research questions stated above by studying the specifics of Colourful as it was conceived of and initially implemented to meet the challenge of an outsized waiting list (See Appendix 1 for more details).

Since the introduction of Canadian Medicare in the 1960 s [14], and across models like the now widely-adopted Patient’s Medical Home (PMH), FPs have been described as ‘quarterbacks’ of primary care teams [15–18] This is to say they have been seen as acting on-the-field to deliver PHC; leading teams of in-clinic providers such as registered nurses (RNs) or medical office assistants; and managing referrals out to specialist care as a steward of the broader system’s resources [17, 18]. However, with the advent of expanded scopes for other health care professionals [19–21]; the increasing complexity of patient needs [2]; and a dearth of FPs [11] these teams led by clinic-owning doctors paid on a Fee for Service (FFS) basis, have come to be regarded as unsustainable and inadequate [1, 12, 22]. In response, polices changing FP funding [23, 24] and pursuing more robust team-based care [25, 26] have emerged, introducing the possibility that FPs may be moving to coaching from quarterbacking.

What follows illustrates an emerging conceptualization of ‘team’ and identifies key areas for policy development in cultural competency, communication, human resources (HR), and professional governance that are likely required for FP ‘coaches’ to become a broadly successful solution to the access crisis. Specifically, we follow the thinking of, and issues encountered by, the Working Group members of the PHC Clinic (hereafter ‘the Clinic’) who were responsible for designing and implementing Colourful (see Appendix 1 for more details).

## Methods

An embedded action researcher from the University of Calgary (AM) was a participant observer at Colourful’s planning and rollout meetings (*N* = 12), supplementing these observations with review of the project’s documents, and interviews with Working Group members. The Working Group verbally consented to the researcher’s presence at the meetings, and was aware that she was taking field notes as well as actively participating in and contributing to the design of Colourful. The field notes from the meetings were used as sensitizing elements for the interviews and analysis that would follow.

Ten Working Group members, from a pool of 16 people, were approached for interviews and 9 agreed to participate, completing a written consent form prior to doing so. Table 1 shows how 11 interviews (9 initial and 2 follow up) were conducted with a range of internal (to the Clinic) and external participants between February 2024 and August 2024. Interviews were conducted online and in person during work hours at participants’ place of work, based on their availability. To protect anonymity, we only ascribe a high-level descriptive title and number to these participants. Their comments have been anonymized and edited to balance privacy and brevity with the provision of important context and detail. Key quotes from the interviews illustrating our analysis are summarized in the Results section of this paper, and can be found in full in Appendix 2.

**Table 1:** Interview recruitment & participation

Work Title	Interview Request Accepted	Interview Completed (O = Online; IP = In Person)	Follow-Up Interview Requested	Follow-Up Interview Completed	Participant Codes Used in Appendix 2
Internal - Nurse	Yes	Yes IP			ADMIN1
Internal - Administration	Yes	Yes O	Yes	Yes O	ADMIN2
Internal - Nurse	Yes	Yes O			ADMIN3
Internal – Physician (Late Career)	Yes	Yes O			PHYS1
Internal – Physician (Mid-Career)	Yes	Yes IP			PHYS2
Internal – Physician (Mid-Career)	Yes	Yes O			PHYS3
Internal – Physician (Early Career)	Yes	Yes O			PHYS4
Internal – Physician (Early Career)	No				n/a
External participant	Yes	Yes O	Yes	Yes O	EXT1
External participant	Yes	Yes O			EXT2

Concentrating interviews on a smaller number of content specialists has been shown to be sufficient for attaining data saturation and adequate information power [27]. In the case of the present study, we were able to obtain data saturation with a relatively small sample size because the Working Group was composed of engaged key informants who were amenable to sufficiently lengthy interviews [28]. As embedded action researchers we were able to conduct near real-time checks with our participants, gaining their insights and feedback through an initial report for Clinic consumption, and drafts of the present manuscript. We developed an interview guide based on the broad research questions described above, and our ongoing observations of the Working Group as it accepted its mandate, identified a root cause problem, and designed Colourful as a ‘coaching’ solution to that root cause. The interview guide was used to structure the conversations with participants and can be found in Appendix 3 Table 2.

**Table 2:** Interview statistics

Total Number of Interviews Completed	11 interviews
Longest Length	74 min 53 s
Shortest Length	25 min 06 s
Average	45 min 17 s
Standard Deviation	15 min 10 s

Digital interview recordings were transcribed by a professional service, and then quality checked by the researchers, before being analysed using an interpretive description approach [29] with the support of MaxQDA™ software [30]. Like other commercially available qualitative analysis software packages, MaxQDA facilitates the storage and manual coding of manuscripts and so the identification of themes and a broader interpretation of the various participants’ viewpoints. Coding categories were developed, expanded, collapsed, and checked by the researchers iteratively, with instances of disagreement discussed until a consensus interpretation could be reached. Interpretive description focuses on identifying applied knowledge and allows for specific attention to participants’ institutional commitments and perspectives [31–33]. It provides insights not just into areas of commonality but also areas of disagreement among participants, with an eye on unearthing pragmatic suggestions to improve policies and outcomes [29, 34]. As noted, we continued to engage as action researchers with our participants, providing them with initial reports, and then drafts of the present manuscript to check and reflect on. In this way our emerging interpretive description of Colourful’s design was merged back into its rollout, informing how the Clinic implemented on the ground and approached advocacy with policy makers.

## Results

As the Working Group designed and implemented Colourful, our analysis shows its members were thinking about, and working with, three key aspects of teamwork: Becoming a Consultant; Human Resources Challenges; and Change Management Capacity.

### Becoming a consultant

Participants noted that Colourful required FPs to transition into the role of a consultant (B1Q1), overseeing the work of others on the team rather than providing direct care themselves (B1Q2). This shift to consulting work was operationalized as one where Clinic nurses became first points of contact and even ‘quarterbacks’ for patient care (B1Q3). An anticipated consequence of this delegation to nurses was that FPs would be less involved in delivering direct patient care (B1Q4).

Newly empowered quarterback nurses, working at the limits of their traditional scope of practice, were seen as central to patient evaluation, treatment, navigation, and triage (B1Q5). Newly acting as consultant overseers in a collaborative team that divides up care delivery, FPs were positioned as authorities at interdisciplinary rounds convened to keep team members informed and in conversation with one another (B1Q6). Working Group members recognized that the shift was not just potentially destabilizing for FPs (B1Q7) but that most were untrained in how to oversee work that had formerly been theirs alone (B1Q8; B1Q9). As such, adjustments to continuing medical education and medical school curricula, as well as to the educational and regulatory environments of the registered nurses to whom Colourful FPs were now delegating direct patient care, were seen as critical (B1Q10). Specifically, participants identified the necessity of education support and governance reform to clarify FPs’ legal and administrative responsibility for the actions of other team members (B1Q11).

Participants noted that the new role might offer less job satisfaction for FPs (B1Q12) and that the spread of the intervention hinged on independent FP’s willingness to delegate their authority and be trained in a new approach to team-based care (B1Q13). This willingness to delegate was in turn tied by Working Group members to historical norms in the medical profession (B1Q14), a sense of physician superiority (B1Q15), and ‘grumpy’ concerns at a loss of turf and income (B1Q16).

In sum, taking on the consultancy role in Colourful’s new version of team involves FPs delegating significant authority and direct care time to nurses as they become the on-the-field quarterbacks delivering PHC. Technical and cultural training in the new methods and mindsets required to oversee work that was once the exclusive purview of FPs is seen as necessary. This training in shared decision making and responsibility is also challenging given norms and concerns amongst physicians.

### Human resources (HR) challenges

When asked which health professionals should be included on the Colourful team, Working Group members agreed that team composition should be driven by a mix of patient needs and structural features. Patient demographics and health needs, local socioeconomics and clinic resources were seen as important factors in determining which professionals are hired onto the team (B2Q1; B2Q2; B2Q3).

The cost of hiring was identified as a barrier to creating the preferred PHC team (B2Q4). Even with adequate funding available, finding the necessary trained professionals to fill the roles may prove difficult, with the lack of availability of Nurse Practitioners and Physician Assistants specifically noted (B2Q5; B2Q6). Once the team was formed, awareness of scopes of practice was identified as necessary to the creation of operating procedures to govern and clarify the delegation of tasks (B2Q7; B2Q8; B2Q9; B2Q10).

Although Clinic nurses were seen as possessing the skills to take up on-field quarterback duties with patients, participants described the need for ongoing training to ensure those nurses were practicing at their full scope and accepting the highest possible volume of delegated tasks (B2Q8; B2Q11; B2Q12). This scope-of-practice and medico-legal responsibility training was being created and implemented by the Clinic ‘in-house’ as they were unable to find premade courses to support this kind of role expansion(B2Q13). One Working Group member noted that the Clinic’s nurses came to this bespoke training from a different cultural and operational baseline than most other nurses in the province would. The effort and resources required to bring not just nurses, but all team members to that baseline were major considerations for future spread and scale (B2Q13; B2Q14). Alongside the creation of in-house training programs, the Clinic planned to develop standard operating procedures and protocols that would support non-physician team member competencies and allow FPs to off-load more tasks (B2Q15). Working Group members noted that existing legislation limited nurses’ abilities to take over certain ‘quarterback’ tasks. Specifically, they identified the inability to sign off on reports (B2Q16), write requisitions and approve labs (B2Q17) and a lack of prescribing authority (B2Q18) as legislation limitations on nurses’ capacities to ease the FP-access bottleneck.

In summary, moving FPs from quarterback to coach role requires consideration of patient and structural issues as hiring decisions are made. Once hired, the new team members require scope-of-practice training; this training, along with standard operating procedures, will need to be developed locally, and as Colourful-like programs spread they will need to accommodate a range of baseline teamwork and integration practices.

### Change management capacity

Colourful’s design and implementation were sparked by an in-Clinic mandate (see Appendix 1) and seen as part of an obligation to the Clinic’s local community (B3Q1; B3Q2). Beyond achieving local access improvements, Working Group members saw Colourful as an opportunity to be innovative (B3Q3), to try something ‘outside of the box’ (B3Q4) and redefine what PHC more broadly could look like (B3Q5;B3Q6).

When envisioning project scaling beyond the Clinic, participants described how external implementation would require more than just sharing the model. Culture work (B3Q7) aimed at shifting mindsets and establishing literacy in the Clinic’s approaches to teamwork as well as the capacity to assess local readiness for change (B3Q8) were identified as necessary. Spreading Colourful would also require additional funding, a challenge that even the alternatively financed Clinic had overcome by rerouting money away from the normal operations budget to cover startup costs (B3Q9). Specifically, Working Group members felt it was necessary to secure funds to support change management and implementation (B3Q10;B3Q11) while safeguarding regular ‘day to day’ practice (B3Q12) and the financial stability of the Clinic (B3Q13).

In summary, Colourful’s creators see it as both an obligatory response to local access issues and a potentially scalable innovation in how team-based PHC is done. For this spread and scale to occur a range of cultural, change management, and financial adjustments that go beyond the mechanics of the quarterback-to-coach shift in FP status must occur.

## Discussion

With early work on the composition and scope of PHC teams emerging around the world [35–40], our findings document how Clinic team members are navigating a transition from on-the-field ‘quarterback’^14^ to consultant ‘coach.’ In this way, the deployment of Colourful to solve the Clinic’s local version of a Canadian PHC access crisis, becomes an opportunity to identify broadly applicable policy considerations. At a time when the government of Alberta – also seeking to improve access – is expanding a version of the Clinic’s alternative payment model to the rest of the province [41–43], the team’s insights into the scope-of-practice training and new operating procedure development that are required to accommodate integration and teamwork are particularly timely. As the province expands the payment model beyond the Clinic, Colourful’s physician-led approach to team-based care may well be poised to scale, but our findings highlight the importance of not just finances, but also cultural and technical capacity if that spread is to occur.

### Cultural capacity

The Colourful team’s recognition that FPs’ mental models need to shift for the new consultant coach role to succeed aligns with both Cronholm [44] and Glazier’s^1^ findings that historical approaches to guarding medical turf must be set aside. Participants recognized that their ‘team-culture’ baseline and openness to innovation were not only unique, but likely more advanced than other clinics, suggesting a broad need for policies supporting competency in the cultural values and norms of effective teamwork. From this baseline, and echoing recent findings on optimal PHC teamwork [45], the Colourful team emphasized the importance of creating opportunities for all team members not just to communicate about clinical topics, but to enact the new ‘coaching’ cultural values of shared decision making and responsibility. The literature suggests that shared clinical decision making [46], autonomy and shared leadership [47] and nurturing a positive team culture [48] all create better team dynamics and reduce burnout. An important consideration for those who design initial and ongoing professional education would be to create enculturation practices that can rewire team members’ thinking to reject traditional ‘doctor with helpers’ quarterbacking and embrace innovative ‘consultative’ coaching.

### Technical capacity – communication & HR supports

Alongside these cultural changes, the Colourful team highlighted the importance of frequent intra-team communication, confirming findings [46, 47] that show such interactions are critical for successful interprofessional collaboration. They also noted that FPs may lack the skills to: hire and coach new team members; oversee those team members in systematic ways; and manage change. At a time when top-down legislative action to enforce interprofessional collaboration has proved ineffective [5] and centralized wait lists for patients wishing to attach themselves to FPs are receiving mixed reviews [49], Colourful was developed locally with attention paid to the demographics and needs of patients on the Clinic’s waiting list. While the majority of provincial policies support this grassroots approach, they also tend to lack details on how local teams can best be composed to meet the needs of target populations [10]. In this sense, further research on PHC team development [50] is likely required to support emerging ‘coach’ teams as they assemble the right mix of members. Even more pragmatically, Colourful’s experience suggests that human resources (HR) supports for those local teams to find, hire, manage, systematically debrief [50], and retain the right qualified personnel are likely requirements for them to succeed and scale.

### Technical capacity – local & provincial governance

The Colourful Team’s experience similarly suggests that education on how to clarify and operationalize the legal and ethical responsibilities involved in team-based care is an important consideration. The policy implications here have internal local and external governance aspects to them, aligning our findings with those who have suggested that a mix of local and provincial-level policy supports are likely required [10, 51]. At the local level, spreading the Colourful model will require bespoke in-house policy and education work across a range of clinical settings. As before, PHC team members cannot be assumed to already have the skills to work collaboratively or that co-location itself creates teamwork [47].

In respects to external governance issues, Colourful team members highlighted how the currently legislated scope of RN practice prevents the delegation of certain ‘quarterback’ tasks from FPs to RNs, limiting the model’s capacity to improve access. In this light, professional associations and policy makers will want to consider adjusting scopes of practice to support the spread of Colourful-like models. Given recent policy moves to grant a range of non-physician providers expanded scopes of practice in PHC, there is considerable pressure for further research and consideration of how these non-physician ‘teams’ might, or might not, work alongside FP ‘coach’ models like Colourful. Specifically, nurse practitioners [52], pharmacists [20, 21]and optometrists [53] have acquired PHC provision capacities and it is unclear how these parallel but presently unconnected care streams can, or ought to be, integrated.

### Limitations

As a qualitative observational study, our data are derived from a specific location at a particular moment in time. Our analysis seeks to transcend these specifics and draw out broader lessons, but that analysis has, inevitably, been shaped by our own biases as social scientists. Although we have followed best practices in studying the context of a healthcare improvement intervention [54] - refining our interpretation of the data by consulting with our participants – our findings may not generalize to other physical clinics, cultural settings, and policy or finance programs. This is particularly true given Colourful’s focus on registered nurses when a range of other allied health practitioners are also operating on, or nearby, PHC teams. As such, further localized assessments aimed at precisely calibrating policy supports are likely necessary.

## Conclusion

Payment model reform is a necessary but insufficient step as health systems seek to improve PHC access by shifting FPs from quarterback to coaching roles. Other key considerations for policy makers as these new teams emerge include dedicating support and resources to build cultural competency and technical capacity. Initial and ongoing educational focus is required to flatten traditional interprofessional hierarchies and enable the communication required for successful teamwork. Similarly, resource and governance supports that bolster FP coaches’ HR skills, ensure an optimized interprofessional mix, and don’t just train team members, but empower them to renegotiate scopes of practice at clinical and provincial levels, will contribute to successful scaling of innovative models and improved access to PHC.

## Data Availability

The datasets used and/or analysed during the current study are available from the corresponding author on reasonable request.

## Appendix 1

### Project colourful and its context

In the spring of 2023, the full-service PHC Clinic where Colourful was initiated had a waitlist of over 4,000 people hoping to attach themselves to FPs. Whereas the vast majority of clinics and FPs in Alberta have, up to 202543, billed the government payer on an FFS basis, the Clinic has been one of a small group operating under a unique capitation funding model. Under the terms of a special letter from the province’s Minister of Health, the Clinic has been paid annually on the basis of the number of patients that are rostered or attached to its FPs. In the more than two decades that the alternative payment model [51] has been in effect, the Clinic has gained a reputation for its innovative ability to operationalize the team-based care concept. It has hired RNs in-house, and extensively leveraged the multi-disciplinary allied health resources of Alberta’s Primary Care Network (PCN) system [18]. 

Given a mandate by the Clinic’s Board of Directors to reduce the waitlist, a Working Group of internal and external participants was created to brainstorm, design and implement a response As this suggests, the Working Group was self-organizing and developed both its own understanding of the root cause of the waitlist, and how best to solve that root cause with increased teamwork within the physician-led capitation funded context of the Clinic. Their collective work would eventually be named Project Colourful. Internally, the Working Group was composed of: Senior, Mid-Career and New Hire Physicians; as well as the clinic’s Medical Lead; Executive Director; Nursing Lead; IT Director; Communications Director; and a Human Resources Director. A Patient Advocate was recruited from the waitlist and also sat on the Working Group. Externally, a director from the PCN to which the clinic belongs; a Project Manager; and a qualitative health services action research team from the University of Calgary were involved. The Working Group began their planning in August 2023 and the project was first implemented in February 2024.

In seeking to reduce the waitlist, the Working Group’s root cause analysis determined that FP availability was creating a bottleneck which impeded access for patients waiting to attach themselves to the Clinic. Colourful’s innovative approach to eliminating the bottleneck was to empower other in-house health professionals to become the first point of contact for waitlist patients. These non-physician team members were to assess and treat patients according to their professional scope of practice, while under the supervision of a ‘responsible’ FP. This meant that FPs would be both quarterbacks on the field, dealing directly with their own patients, and supervisor coaches working with allied health colleagues also employed by the Clinic as they acquired patients off the waitlist. The initial implementation of Colourful focused on deploying in-house RNs as first points of contact, with the longer-term goal being to expand the team to other allied health practitioners. A physician’s assistant was added to Colourful in July 2024. 

The Working Group sought out feedback on Colourful’s rollout from both colleagues at the Clinic, and patients leaving the waitlist to become attached to a non-FP as their first point of contact. Responses to these surveys were used to course correct the intervention’s deployment, and to gauge patient satisfaction.

## Appendix 2

### Full quotes summarized in the results section

**Table 3:** Interview quotes referenced in the Results section “Becoming a Consultant”

Quote #	Identifier	Quote
B1Q1	PHYS3	So, the idea [for Colourful] was we would be consultants. [The consultant role] was another way of …helping look after patients who need care.
B1Q2	ADMIN2	But I would say [the physician] in many cases acts as a consultant and empowers the team to provide care versus providing care directly. And that’s been a very important paradigm shift. And again, I think something that when I look at where the health system is going and when I look at that physician shortage, I feel like [the broader system is] going to need to move there.
B1Q3	PHYS1	[As Colourful has evolved we’ve come to say:], “Let’s position nurses – who have a broad scope of practice and sort of expertise – [as] those people who are quarterbacks, health managers, advocates.” Whatever word you’d like to use. And then [those nurses would be] surrounded by team members who can be accessed and can be involved in care, [according to] the patient’s needs at that time.
B1Q4	EXT1	When we look at what [a nurse] can do, I think [the physician] will become a lot less involved in [care and when they do it] will be very specific. [As we’ve set up Colourful] the physician or most responsible provider – if it’s an NP, physician assistant, whoever it is –can delegate some of that to the RN.
B1Q5	PHYS2	So, the role of the nurse is going to be the secret sauce…[Colourful nurses] will be definitely pushing the maximum end of their scope [of practice], around independent patient [*pause*]… What’s the word? [*pause*] Evaluation. Potentially [also], treatment and advice. Within scope. Potentially in-clinic procedures, like pap smears and women’s wellness checks. Navigation as well. Triage: [the nurse] might be the first point of clinical contact [and so they will] ensure that the patient is connected to whoever the next right provider is, if it’s not [the nurse themselves]. That’s how I see it. …[All of us] independently working to full-scope, but definitely collaborating, as a team.
B1Q6	PHYS4	As part of [Colourful], we’ve designed this kind of round system where we review regularly because we’re starting this more team-based approach and dividing up care. We wanted to make sure that we were sharing learnings and bringing up cases [that needed attention].
B1Q7	PHYS2	But I foresee, under Project [Colourful], we’ll learn how to look after our large panel, together, as opposed to, “My patients.” And I know that we’re experiencing a bit of grief around losing the potential for being that most important person to the patient, but there will be people that need us. And there are ways to maintain a relationship.
B1Q8	ADMIN2	[the FP is involved in direct care] when the patient’s needs require that level of expertise, but outside of that, the physician is really just coordinating and providing guidance. And I do think, again, if we’re going to expand capacity in our community, we’re going to need to teach family physicians that their roles need to evolve.
B1Q9	PHYS1	Wouldn’t it be wonderful if we started to train our physicians early to think about interdisciplinary care to see their role as manager as much as an individual provider?
B1Q10	ADMIN2	We’re going to need to train physicians to be consultants, we’re going to need to train them to do casework and case reviews with team members. And we’re going to need to make sure that those team members have skill sets and scopes through their regulatory bodies that allow them to deliver safe and effective care in the community.
B1Q11	PHYS2	Since [I presented Colourful at a conference] I’ve had one or two emails [from attendees saying:] “I brought this idea back to my [external clinic], and all the doctors are worried about being responsible for the other team members’ work, or accountable and responsible.” So, there’s some myth-busting that has to happen around interdisciplinary team-based care.
B1Q12	ADMIN2	And I’m curious whether there’ll be as much job satisfaction for physicians who probably spend less time delivering care and more time empowering and leading team
B1Q13	EXT2	Not all physicians use their team in [the manner of a consultant] way, even though that’s well within their scope of practice. The physician’s decision [to move into the consultant role] and to some extent the willingness of the physician to engage in full scope team-based care is dependent on that individual physician
B1Q14	PHYS1	I was trained in the good old days where doctors were gods and they knew everything and they would tell everybody else what to do. And so there’s still, I think, that DNA or aberrant genes floating around the medical gene pool. And I think that is especially [relevant] as we’re [starting to work] to the full scope of [everyone’s] practice. There’s this paradoxical paranoia that other [FPs] feel [and it has them thinking], “Well, I’ll be replaced!”
B1Q15	EXT2	And we still have some physicians in the mental model of, the team shouldn’t be doing anything except what I ask them to do, like physician orders. Which in my view is not true team-based care
B1Q16	PHYS4	Sometimes if you read the news, you hear about these physicians who are very grumpy, that [other health professionals] are kind of encroaching on their patients

**Table 4:** Interview quotes referenced in the Results section “Human Resource Challenges”

Quote #	Identifier	Quote
B2Q1	PHYS2	So, I think, obviously [deciding which professions are on the care team] would depend on the needs of the population or the demographic and health needs of the population, social determinants as well.
B2Q2	PHYS4	I think, and we had lots of discussions on this as well, but I think [who is on the care team] probably depends on the patient panel. And I think depending on where you are in the province and who you’re rostering and what the socioeconomics are and the resources and all of that would change your team
B2Q3	PHYS1	So coming back to what’s the dream team, I think [who is on the care team] depends on your patient population. So I think general principles though, we need more physician assistants, I think we need to clearly define the nurse practitioner role within the team and part of that may be dependent on the population and part of it on the nurse practitioner. Obviously lots of nurses, mental health specialists, and I think the bringing in the physio is actually quite cool too.
B2Q4	PHYS2	Part of the problem [when determining who is on the initial care team for Colourful] is upfront cost, so it’s just a dollars and cents thing, around those. Well, NPs, anyway, are, as a more expensive healthcare provider. And maintaining sort of the lowest cost provider, working to their fullest scope, as a bit of a principle. So, sad, but true, in publicly funded [health systems]. You know? Someone’s got to pay for it.
B2Q5	ADMIN1	And [hiring other professionals to work on the team] wouldn’t necessarily be fixed with funding, though, ‘cause we may not physically have the bodies to do that [Nurse Practitioner] and [Physician Assistant] piece.
B2Q6	PHYS4	I do think though finances are a limiting factor [in hiring a care team], and then also hiring the right people, because we have been thinking about [Nurse Practitioners] and [Physician Assistants] for some time and finding these people is not necessarily really straightforward.
B2Q7	PHYS4	Yeah, so we’ve had lots of ideas about [who is the most responsible provider] on the design team. I think most of the colleges for the pharmacists and the nurses and the physician assistants and the NPs, they do have pretty clear kind of scope. And so our thought is we should be using these different practitioners within their scope. And then some thoughts on, again, does everybody know kind of, “Hey, this is in my scope or out of my scope”? And so some kind of internal assessment processes to understand that.
B2Q8	ADMIN3	[The physicians] are very supportive of us kind of doing any additional training that we’re interested in and seeing which ways it’s beneficial for us to expand our scope. So we’re kind of, I guess seeing what works and what doesn’t, there’s certain things that would be more efficient for us to do [and] things that wouldn’t be as efficient for us to do in the RN role, just depending on whether a physician needs to be directly involved or not.
B2Q9	ADMIN1	So if the patient has a need it’s looking at, okay, which of our team members is best suited from a scope perspective to be able to take care of that?
B2Q10	PHYS3	I’m just thinking about the categories of people [and how they would be organized and seen in Project Colourful]. So, we have people, patients I mean, who may just be RN attached because they’re really straightforward and nothing to get concerned about consulting occasionally. And there’ll be people who are [Nurse Practitioner] or [Physician Assistant] attached [pause] who are a bit more complex but not quite as complex. And then, the really sick, [those patients are] over here with [the physician], so we would have some protocol.
B2Q11	PHYS2	I do think that we’re on track with up-training our registered nurses for more mental health support.
B2Q12	ADMIN3	[Project Colourful] has been starting to intake patients and starting to grow our patient panel in the last few weeks. So my kind of day-to-day on the team right now is either doing training that will be used in the future just to expand my scope and then also doing those patient intakes.
B2Q13	ADMIN1	I can research [the skills] needed and we can look up the theory and learn that together, but our nurses are learning from the doctors or from [the clinic] nurse practitioner, for some of the women’s wellness things. And so you need to have [the resources and ability to train] as well, because there isn’t somewhere where we can send our nurses or programs available, ‘cause it doesn’t exist. We’re the first ones doing it.
B2Q14	ADMIN1	And not to say it couldn’t be there, but the groundwork [for nurses in other clinics to work in an environment similar to Project Colourful] wouldn’t be the same, to my understanding, just for a sheer fact of what is available for resources for them for sure. So I think it’s not that [Project Colourful in other clinics] couldn’t happen, but it would take a lot more time. ‘Cause when we have nurses start at [the regular practice of the clinic], like even myself, usually no one has come from anything similar to this in all different backgrounds.
B2Q15	EXT1	There are [patient interactions] that would maybe not have been done by one of the nurses previously because they didn’t have the skills experience, but they’re learning, and the protocols are being set up so that the physician is delegating those [tasks] to those team members. So again, if the physician is delegating and there’s a protocol that the nurse is following, they don’t need to go back to the physician unless something occurs that takes them outside of that protocol.
B2Q16	PHYS3	We can get a nurse to do a PAP, but she can’t sign off on a mammogram report, really? Especially a completely normal mammogram report. Anyways, so somebody needs to do that, otherwise [the physician] is going to have way too freaking much work to do that’s tedious as all heck.
B2Q17	EXT1	I think [the working group] has been exploring a lot about scope of practice and these sorts of things for the roles, and it really does seem as if the RNs have an immense scope of practice, a lot of which hasn’t been tapped into up till now. [But] there are still some sorts of decision making, signing, approving labs, making requisitions, these sorts of things that are beyond what is the scope of the RN.
B2Q18	ADMIN1	But it’s having a [team member other than a nurse], I really feel like that’s a crucial piece to being able to really make this a larger-scale, scalable thing, is just my guess. But I don’t know, ‘cause we haven’t even started the actual work with [the] RN position yet, but I have a sense that to truly help support the physician from that medical-legal piece and that kind of MRP-related, medically the most responsible provider, that really having an allied person that has that diagnosing, prescribing and ordering capability is what will be a big link.

**Table 5:** Interview quotes referenced in the Results section “Change Management Capacity”

Quote #	Identifier	Quote
B3Q1	PHYS2	We have a wait list of 4,000 people. One of our missions is to [provide] better health for the community, “What do we do for the community? How can we expand the care that we can provide?” We know, in the given climate, in our traditional [clinic], there isn’t capacity, necessarily, to take on 4,000 patients, so, “Can we test something different?”
B3Q2	PHYS1	Yeah, I think [Project Colourful] started with a mandate that the board gave us a year and a half ago to say we need to do more to support the community. Our value statement talks about giving to the community, our mission statement, I should say. And we say, “Well, we think we’re doing a pretty good job providing a medical home for patients who are attached to us, but we’re really not helping the community and the shortage.” So we’ve started to explore different ways that we could leverage the expertise that we have now and essentially provide a medical home for more patients.
B3Q3	ADMIN2	[The clinic] is a highly progressive group. Very committed to quality, very committed to innovation, and very committed to their community.
B3Q4	PHYS1	I think that we can be dreamers, and I think there was a sense that we wanted to try something that was different, that was outside the box, that was innovative, because, again, that’s part of our core.
B3Q5	ADMIN2	So [Project Colourful] was really dreamed up in our business strategy as a way to do a couple things, makes [the clinic] more sustainable to actually test the boundaries of what primary care and team-based primary care could look like, recognizing that we have to redefine what care looks like to have a sustainable health system in the future, and then to be able to scale and spread that to our community partners and to measure the impact that has, not only on a patient’s experience and journey within [the clinic], but within the context of the health system.
B3Q6	PHYS1	And the other thing we thought with [Project Colourful] is that this felt really risky, it felt really out there. It felt like something that would give us a lot of arrows. So we thought let’s widen the tent as much as we can, let’s take the arrows upfront, understand what some of the concerns are, where the supporters are, and see if we can do this in a way that is building momentum of a number of people rather than creating something and saying, “Look at our beautiful creation, you should do this.”
B3Q7	PHYS2	I think [helping others implement Project Colourful is] a bit more than a friendly phone call, because there’s lot of culture work that happens, that [the clinic], I think, at times, takes for granted, that we have in terms of readiness for a novel idea, that others might not be ready for, so I think it’s both [culture and support from the clinic].
B3Q8	ADMIN2	I think it’s less about me offering a template [for Project Colourful] that somebody can plug into their clinic, and it’s more about literacy, education, advocacy, shifting the mindset, assessing readiness for change because if those things aren’t in place, it actually doesn’t matter what we give to somebody, it can’t be executed the way it was intended to or the way that has an impact on our health system and on the patient journey.
B3Q9	EXT1	I think the biggest weakness for [Project Rainbow] is probably, as I mentioned, just that lack of upfront funding for it, that they’re having to fund it out of their existing clinic operational budget. There’s no additional money for it.
B3Q10	PHYS1	We’ve been very sensitive around the financial part because I think the owners, the message that they said was, “All this sounds good in theory, but how much is this going to cost us?” And early on, we did look for alternative funding. And I’m sort of proud to say when that didn’t come up, it didn’t change the passion to go forward. So what I can absolutely say to you is the motive for this was not to make more money, but we also wanted to make sure that we weren’t losing our shirts in doing it. And I think we’ve struck a balance. It’s affected how our change management has gone, but I think we’ve been pragmatic around it.
B3Q11	ADMIN2	So again, I think in the context of financial viability, if money were no object, we would’ve designed [Project Colourful] one way, but we’re a business, right? It’s really interesting. I presented at a conference once and somebody stood up and said, “Wow, you run your clinic like a business,” And it is a business.
B3Q13	PHYS2	One of the goals is, there’s a group of owners that are responsible, and there is an expectation of cost recovery, if not profit friendly.

## Appendix 3

### Interview guide

#### Interview goals

Understanding the origins, goals, and potential for expansion of [the Clinic’s] teamwork focused *Project [Colourful]*

#### Script

Thank you for agreeing to participate in this study. The purpose of our time today is to better understand:

- Where Project [Colourful] came from;
- What explicit and implicit goals for teamwork and service delivery Project [Colourful] is pursuing;
- Any knock-on or external policy implications that are inherent in those teamwork and service delivery goals; and finally
- Where and how Project Rainbow might expand beyond its origins in the [Clinic], across the [local area], and across the Province [of Alberta].

In asking questions to get at these topics, I’m likely to ground our conversation in the details of prescribing generally and anti-biotic prescribing specifically. In this way I’ll be using prescribing as a touchpoint – a concrete moment of action and interprofessional interaction – when we talk about the teamwork innovations and service delivery goals that are being pursued with Project [Colourful]. The goal here is to talk pragmatically about how the primary care team is, or isn’t, being reimagined through Project [Colourful]. I’m particularly interested in understanding how the Project [Colourful] model differs from, or is similar to, the teamwork and service delivery aims of: normal [Clinic] operations; and normal operations in the wider Fee-For-Service primary care system.

To get at these teamwork and service delivery issues, I will ask you some open-ended questions and you are welcome to answer any way you like. As you answer, I will be taking notes of things you mention that I may want to explore further. There are no right or wrong answers, and you are free to decline to answer any questions that you don’t feel comfortable with. You decide how much you want to share. I would also like to remind you that everything said during our interview will be kept confidential. 

The transcripts of this interview will be de-identified for confidentiality purposes. This isn’t a guarantee that someone from your work or personal circles will not be able to identify you in a final published manuscript, but all references to people or places will be removed. Our academic goal here is not to report on the details of Project [Colourful], so much as to draw out broadly applicable considerations for improving Albertans’ and even Canadians’ access to multi-disciplinary Primary Care teams. Please also know that you can take a break or stop the interview at any time.

Do you have any questions before we begin?

With your consent, I am now going to start recording and begin the interview.

1. Can you please give me the cocktail party version of yourself? Where did you train; how did you end up in Primary Care; how long have you been in your present role; and any relevant roles you might have held prior to it.
2. Do you have a preferred descriptor we could use when drawing on these interviews?
3. Can you describe to me how you came to be involved with Project [Colourful] and what you do for the project now?
  - Where did Project [Colourful] come from?
  - Who was involved in its development? How were participants selected?
  - Which stakeholders were involved? Was there anyone specifically, or accidentally, not included? If so, why?
4. What kind of problems, in your mind, is Project [Colourful] intended to solve?
  - Is the problem a specific experience here at [the Clinic]? Is it broader than [the Clinic]?
  - Are there any other goals of the Project (internal, external, etc.)?
5. I would like to focus back on that broader Teamwork Innovation model and talk about how Project [Colourful] might be expanded. What does that expansion look like:
  - In [the Clinic]?
  - In the [local region]?
  - Does this project fulfill the objectives of the […]PMH model? If yes, why? If not, how is it different?
6. Now that we’ve talked about the explicit and implicit goals, I’m interested in what you think success looks like for each of them. Can you tell me about that, what success looks like for each of those goals we just talked about?
  - What happens to Project [Colourful] once each of those goals is met?
  - Are there any bigger policy consequences if the project proves successful? What policy changes may be required for the project to find success?
7. Does the unique funding model of [the Clinic] play a role in the implementation of Project [Colourful]?
  - If so, what is that role?
  - Does this relationship between your funding model and Project [Colourful] limit expansion into other practice environments?
8. What role does interdisciplinary collaboration play in Project [Colourful]? What role does non-physician independent practice play in Project [Colourful]?
  - Is that role different to what occurs in normal [Clinic] operations?
  - Would a patient notice that they’re in the [Colourful] stream or out of it? What are the key similarities and differences in how a patient will interact with the different team members in the [Colourful] stream?
9. To provide me with a better understanding of what daily practice looks like in the Project [Colourful] setting, I would like to discuss an example. Antimicrobial Resistance is a growing problem, and the literature points the finger at primary care for poor prescribing habits. How, if at all, might the kind of teamwork changes – the new collaborations or interaction structures -- we were just talking about shape improvements in antimicrobial stewardship generally, or antibiotic prescribing specifically?
  - In the setting of Project [Colourful], with many practitioners who can assess for and prescribe antibiotics, would there be focus on consistency amongst practitioners? In the context of antibiotics, would there be a role for policies, guidelines or second opinions?
  - What would happen in the case of a disagreement over treatment?
10. It seems to me that patients will inevitably utilize other health professionals outside of Project [Colourful]; such is the case with the antibiotic prescription and the dispensing pharmacist. As pharmacy, physiotherapy, psychology, and other practices currently exist outside of the clinic, how is the Project [Colourful] move to bring these services together in a “one roof” model an innovation that improves access?
  - What are the benefits of the “one roof” model? What are possible risks of the “one roof” model?
  - There is a school of thinking in primary care policy research that suggests enhanced scopes of practice, and the expansion of independent practice for non-physicians are ways to increase access. Project [Colourful] seems to be consolidating existing scopes of practice and, by requiring that a patient be assigned to a physician for funding purposes, it seems to circumscribe the practice of non-physicians, drawing them under the authority of a physician. Am I right about this? If not, can you please clarify for me?
  - Could a MRP (Most Responsible Provider) ever practice outside of a physician clinic? Can you please share your thoughts on why or why not.
11. Is Project [Colourful] a pilot project?
  - If no, why? If yes, what, in your mind, sets this pilot project apart from the ones that us Canadians are so fond of launching?
12. Do you have any closing thoughts about Project [Colourful] that you would like to share?
13. Is there anyone else I should be talking to? Are there other individuals or groups that you think I should talk to about this work?

## Abbreviations

FP: Family Physician
FFS: Fee for Service
HR: Human Resources
RN: Registered Nurse
PHC: Primary Health Care
FFS: Fee-For-Service
PMH: Patient Medical Home
PCN: Primary Care Network

## Footnotes

1 A pseudonym for the project created to provide anonymity to participants.
